# Giant Gastrointestinal Stromal Tumors of the Stomach Successfully Treated With Laparoscopic Resection: Case Report and Literature Review

**DOI:** 10.7759/cureus.13584

**Published:** 2021-02-27

**Authors:** Abbas Mohamed, Turki Al Qureshi, Saeed M Rakha

**Affiliations:** 1 General and Laparoscopic Surgery, National Guard Hospital, Al Madinah, SAU; 2 General Surgery, National Guard Hospital, Al Madinah, SAU; 3 General Surgery, Ministry of National Guard - Health Affairs, Al Madinah, SAU

**Keywords:** gastrointestinal stromal tumors, open resection, laparoscopic resection

## Abstract

The stomach is the most common site of gastrointestinal stromal tumors (GISTs), representing 60% to 70% of all GIST tumors of the gastrointestinal tract. Gastric GISTs are usually asymptomatic discovered incidentally during endoscopic or radiological investigations. A small percentage may present with melena, hematemesis, and anemia due to recurrent bleeding. We report a case of a giant gastric GIST presented with anemia, that successfully treated with laparoscopic resection.

## Introduction

Although gastrointestinal stromal tumors (GISTs) are the most common tumors of mesenchymal origin of the gastrointestinal tract, they are rare constituting less than 1% of all gastrointestinal neoplasms [1]. In the past GISTs were treated with open surgical radical resection because of their malignant potential. At present, due to a better understanding of the tumor biology and introduction of tyrosine kinase inhibitors, wide local resection with margins became the principal treatment. The role of laparoscopy in the management of giant gastric GISTs remained debatable because of concerns about intraoperative tumor rupture and the dissemination of tumor cells, however; there is increasing evidence of the feasibility and safety of laparoscopic resection. We present a 46-year-old male who discovered to have a giant gastric GIST on upper gastrointestinal endoscopy performed to investigate asymptomatic anemia. The histopathology and immunostaining of the tumor biopsies confirmed the diagnosis of GIST of the spindle cell variant. He was successfully treated with laparoscopic wedge resection. We also searched the literature for the role of laparoscopy in the surgical management of giant gastric GISTs.

## Case presentation

A 45-year-old male was admitted to the medical ward at the National Guard Hospital, Al Madinah, KSA, for investigations of asymptomatic anemia, which was discovered incidentally on a routine medical checkup. He had no symptoms of chronic dyspepsia, epigastric pain, and no history of alteration of bowel habits, rectal bleeding, or loss of weight. Clinical examination was normal apart from paleness.

Laboratory investigations showed hemoglobin about 9.4%, hematocrit 30.2%, MCV66, MCH 20.4, and MCHC 31%. The white blood cell count was 8.9 K/mm^3^ with 70% neutrophils and 16.5% lymphocytes. Other blood tests, including urea, electrolytes, liver function test, and coagulation profile was normal.

He had an upper GI endoscopy, which revealed a sizeable exophytic tumor arising from the greater curvature of the stomach occupying almost all the body of the stomach and part of the fundus highly suggestive of GIST tumor. The pyloric antrum and the first part of the duodenum were not involved by the tumor. Multiple biopsies were taken and sent for histology.

The histopathology of biopsies showed spindle shape tumor cells with eosinophilic cytoplasm and syncytial growth pattern. The nuclei were noticed to be elongated with inconspicuous nucleoli exhibiting marked nuclear pleomorphism and hyperchromasia. The histological appearance was suggestive of the spindle cell variant of GIST. The differential diagnosis includes leiomyoma, schwannomas, inflammatory myofibroblastic tumors, and solitary fibrous tumors.

**Figure 1:**
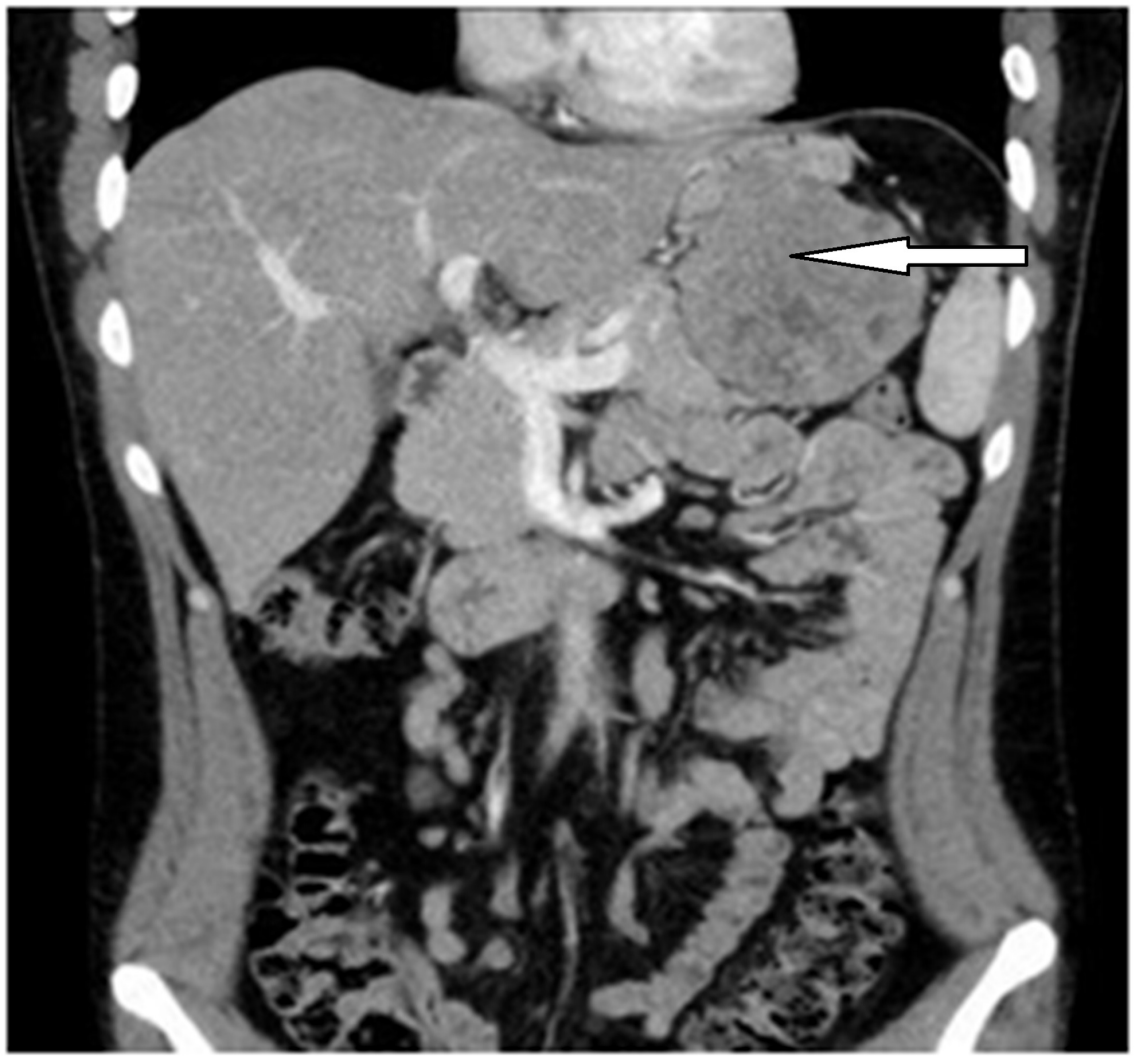
Abdominal CT scan image (coronary view) showing the mass arising from the proximal gastric greater curvature of the stomach displacing the tail pancreas without evidences of pancreatic invasion or hepatic metastasis.

**Figure 2:**
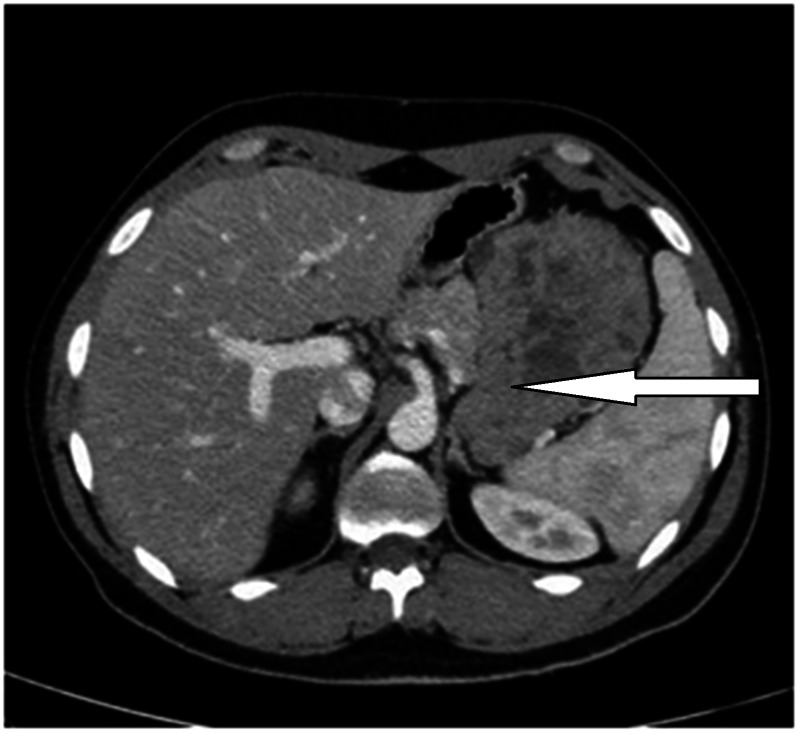
CT scan image (axial view) showing the mass with areas of cystic necrosis, reaching the left adrenal gland and the medial surface of the spleen with no evidences of invasion.

His chest and abdomen CT scan studies showed a well-defined heterogeneous enhancing mass, measuring about 11 x 9 x 8.3 cm. The mass appeared to be exophytic arising from the wall of the proximal gastric greater curvature and showing multiple areas of cystic necrosis. Its inferior part was abutting and displacing the tail of the pancreas inferiorly without signs of pancreatic invasion). The tumor was extending laterally, reaching the left adrenal gland and medial surface of the spleen without evidence of invasion (Figures 1, 2).

The decision for laparoscopic resection was taken on basis of the endoscopic, histopathology, and CT scan findings which were highly suggestive of gastric GIST.

**Figure 3:**
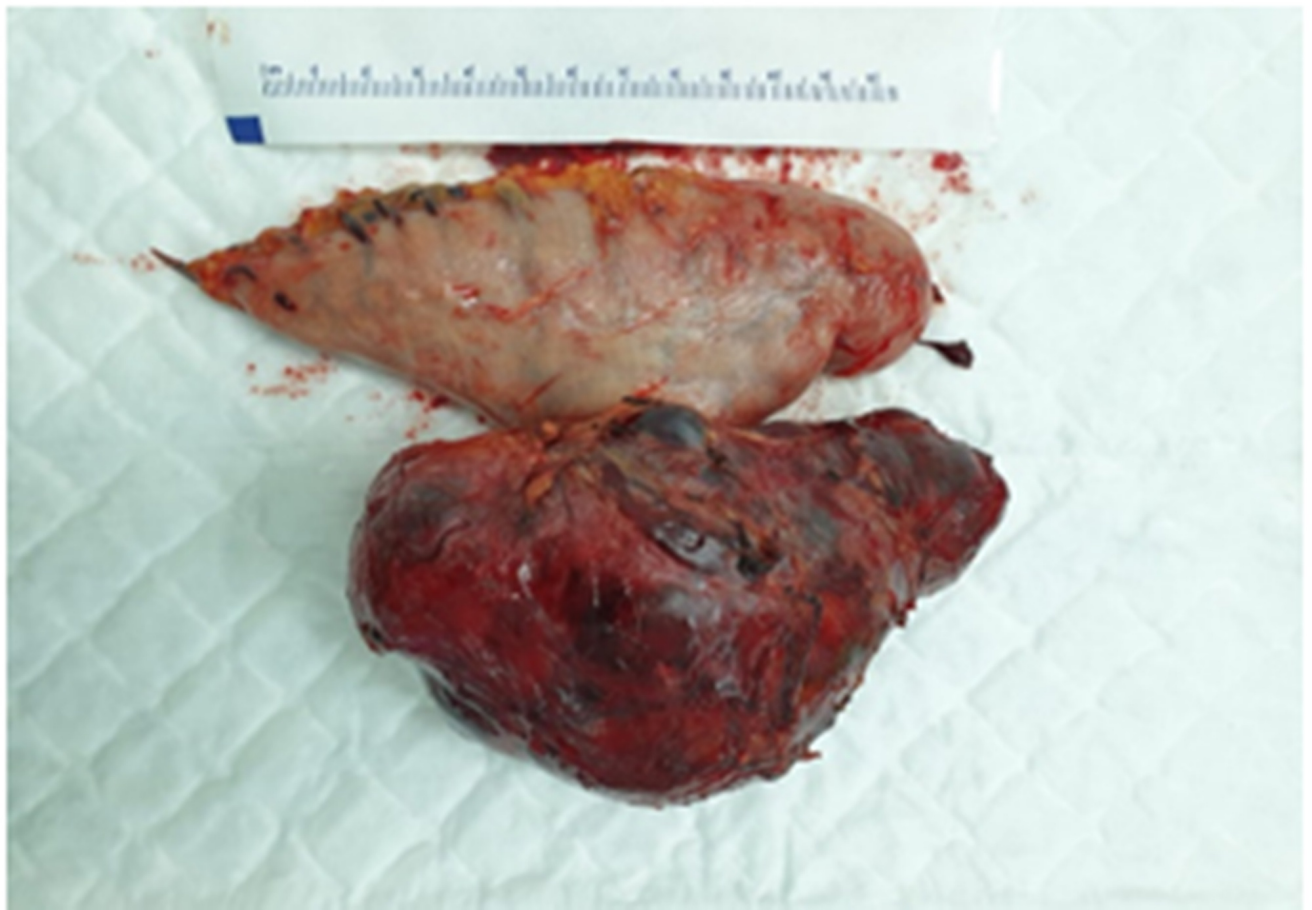
Photograph showing the size of the tumor and its attachment to the stomach.

The surgery was performed laparoscopically by four trocars, two 10 mm trocars, and two 5 mm trocars, which were placed as for sleeve gastrectomy procedure. The tumor was found arising from the greater curvature of the stomach involving about 2 to 3cm of the stomach body wall. It was carefully dissected from the pancreas and resected with a wide clear margin using Endo GIA™ (Covidien-USA) reinforced stapler. The specimen was removed from the abdomen intact through a transverse suprapubic incision about 5-7 cm in length (Figure 3).

Macroscopically the specimen consisted of part of the stomach measuring 12 cm in length and 7 cm in width with an attached mass on the outer surface measuring 13 x 8 x 6 cm in maximum dimensions. On opening the stomach, the tumor was protruding into the gastric lumen on the mucosal side with an ulcerated surface covered with necrotic slough and measuring 3 x 2 cm. It was well encapsulated with no lymph node enlargement.

Microscopically there were spindle tumor cells with eosinophilic cytoplasm and syncytial growth pattern. The nuclei were long with inconspicuous nucleoli. The nucleoli showed marked nuclear pleomorphism and hyperchromasia.

The immunostaining, of the tumor cells, showed a diffuse strong positive reaction with CD34, CD117, DOG-1, and CD 99. There was a negative reaction to SAMA, Desmin, S100, and ALK-1. Both the morphological features of the tumor and the immunohistochemical profile were consistent with the GIST of the spindle cell variant.

## Discussion

GISTs are relatively uncommon constituting less than 1% of all gastrointestinal neoplasms, they are the most common tumors of mesenchymal origin of the gastrointestinal tract [1]. It constitutes about 60%-70% of GIST tumors that occur in the stomach, small intestine (20%-25%), colorectal (5%), and esophagus (<5%) [2]. Gastric GISTs account for approximately 3% of all gastric tumors with only 10-30% of Gastric GISTs behave in an overtly malignant manner [3].

GISTs rarely occur as primary tumors in the omentum, mesentery, or retroperitoneum, but most of the tumors in these sites are metastases from gastric or intestinal primary [4].

GISTs are usually asymptomatic incidentally discovered during endoscopies and radiological investigations. A few cases (25%) present with melena, hematemesis, and anemia due to recurrent bleeding. Other presenting symptoms and signs include early satiety, abdominal pain, and a palpable mass.

The histological features of GIST include spindle cells with eosinophilic cytoplasm and elongated nuclei. The nuclei showed pleomorphism and hyperchromasia. The positive immunoreactivity to c-KIT usually confirms the diagnosis [5]. A small percentage (5%) of the GIST tumors do not show positive c-KIT immunoreactivity, most probably because these tumors harbor mutations in the platelet-derived growth factor receptor. [6]. KIT-negative GISTs can be identified by some other different markers like calcium-dependent and receptor-activated chloride channel protein, (known as DOG1), protein kinase C and carbonic anhydrase [7].

It is important to differentiate GIST tumors from other tumors of mesenchymal origin because of their known resistance to chemo and radiotherapy and their favorite response to the targeted treatment, e.g., imatinib [8].

In the past GISTs were treated with radical resection due to their malignant potential. At present, due to a better understanding of the tumor biology and introduction of tyrosine kinase inhibitors, wide local resection with margins became the principal treatment. 

Traditionally, surgical resection has been achieved through open surgery, necessitating prolonged hospital stays. Laparoscopic surgery is relatively new for GISTs resection; however, multiple studies reported advantages of the laparoscopic approach over the open approach. These advantages include a significantly lower risk of minor complications associated with laparoscopic surgery, as well as a decreased postoperative hospital stay, and early return of bowel functions [9].

The role of laparoscopy in the management of giant gastric GISTs remains debatable due to the concerns about intraoperative tumor rupture and the dissemination of tumor cells. At present, there is no consensus regarding the ideal approach for managing giant GIST tumors.

The current guidelines or consensus from the National Comprehensive Cancer Network (NCCN) and the European Sarcoma Network Working Group, (ESMO) discourage the use of laparoscopic surgery for large gastric GISTs [10].

Recommendations of GISTs consensus conference (2004) accepts the laparoscopic approach for only small GISTs (<2 cm) due to concerns of tumor rupture or spillage [11].

The clinical practice guidelines for gastrointestinal stromal tumor (GIST) in Japan suggest that the laparoscopic resection of gastric GISTs should be reserved for patients with a tumor of size <5 cm and performed only by a skilled surgeon who is familiar with the neoplastic characteristics of gastric GISTs [12].

Recent evidence suggests that prognosis is mainly based on tumor size and histological features rather than the width of resection margins, which makes laparoscopic resection more popular for GIST treatment [13].

The safety and feasibility of the laparoscopic resection of gastric GISTs have been confirmed by several studies [9,14-20]

Zheng L, Ding W, et al. [14] conducted a meta-analysis comparing the surgical and oncologic outcomes of patients with (GISTs) undergoing laparoscopic resection and open resection. They searched Pub Med, Ovid, and the web of science, Cochrane, and Chinese biomedical database. They retrieved seventeen studies that involved 776 patients. Their meta-analysis concluded that laparoscopic resection for gastric GISTs was associated with less blood loss, earlier return of bowel function, earlier resumption of diet, and shorter length of hospital

A similar meta-analysis was conducted by Ohtani H, et al [15] evaluating and comparing the short- and long-term outcomes of laparoscopic resection and open surgical resection for gastric GISTs. They searched MEDLINE, EMBASE, Science Citation Index, and the Controlled Trial Register for relevant articles published between 2000 and 2013 by using the search terms (laparoscopic) (laparoscopy-assisted surgery) (gastrointestinal tumor) (GIST) and "gastric". They retrieved 12 articles that included 644 patients with GISTs; 312 had laparoscopic surgery, and 332 had open surgery. They concluded that Laparoscopic surgery may be an acceptable surgical treatment option compared to open surgery for gastric GISTs.

Lin J, et al [16] in their retrospective study involving 46 patients who were randomized for open and laparoscopic resection of GISTs more than 5 cm (23 laparoscopic vs. 23 open) with mean tumor sizes in the laparoscopic group of 7.2 cm and 7.3 cm in the open group. They confirmed that there were no significant differences in terms of postoperative complications, disease-free survival, and recurrence or metastasis. Metastasis occurred in three cases of the laparoscopic group and five cases of the open surgery group. They concluded that laparoscopic resection for gastric GISTs larger than 5 cm is safe and effective when performed by experienced surgeons.

Similarly Cao, F et al [17] in their prospective study to determine the feasibility and safety of the laparoscopic approach in the treatment of large gastric GIST tumors involving 16 patients who underwent laparoscopic resection for gastric GISTs with a mean tumor size of 7.04 cm (range, 5.2 10.8 cm) demonstrated that there were no tumor rupture, postoperative complications, and local or distant recurrence after follow up ranged between 2 months and 38 months. They concluded that that laparoscopic resection for large gastric GISTs is safe and feasible and should be considered as the standard approach in all cases, irrespective of tumor size or location.

Ortenzi M et al. [18] in their comparative study which included 60 patients with gastric GIST who were randomized for open surgery (22 patients), and laparoscopic wedge resection (38 patients), reported that operation time was significantly lower in the laparoscopic group (82.4 versus 117.8 minutes) and there were no intraoperative or postoperative complications in the laparoscopic group. They concluded that Laparoscopic surgery is a minimally invasive approach to the treatment of GISTs and offers many advantages over the open surgery approach including shorter hospital stay and lower morbidity. The oncological outcomes of the laparoscopic approach for gastric GIST, assessed as tumor-free resection margins and recurrence rate, are comparable to the traditional open strategy.

Chen et al. [19] prospectively studied 214 patients who underwent primary gastric GISTs resection (133 laparoscopic resections, and 81 open resections). They reported that the laparoscopic group had less blood loss and shorter operation time, shorter time to resume fluid diet, faster postoperative recovery, and fewer postoperative complications. They concluded that laparoscopic resection for gastric GISTs resulted in improved short-term outcomes with almost similar long-term outcomes compared with open surgery.

Similarly, De Vogelaere K, et al. [9] in their prospective analysis of 53 primary gastric GISTs resections that were performed in their department (37 patients) by Laparoscopic resections and (16 patients) by open surgical resections, reported that laparoscopic resection of gastric stromal tumors is associated with shorter operation time, a shorter hospital stay, and a lower recurrence rate compared to open resection.

Takahashi T et al. [20] in their retrospective study involved twenty-seven consecutive patients who underwent resection of gastric GISTs >5 cm randomized for open and laparoscopic resection concluded that laparoscopic surgery is feasible, safe and oncologically acceptable, and comparable to open surgical resection in term of the mitotic count, operative complications and the disease-free intervals.

It seems that regardless of the surgical approaches for resection of GISTs tumors, the golden rules are to avoid violation of tumor capsule as it is believed to be associated with tumor’s cell seeding and local recurrence and to ensure complete resection with negative microscopic margins to reduce locoregional recurrence.

## Conclusions

GIST tumors are the most common mesenchymal tumors of the gastrointestinal tract with 70% of them occurring in the stomach. In the past, radical resection was the classical surgical approach for GIST tumors, but with a better understanding of the tumor biology and the introduction of tyrosine kinase inhibitor drugs, wide local resection with negative margins became the principal treatment at present. The role of laparoscopy in resection for giant gastric GISTs still remained debatable. At present there is no consensus regarding the best approach for managing GIST tumors (> 5 cm); however, there is increasing evidence that laparoscopic resection is feasible and safe regardless of tumor size if performed by skilled surgeons who observe the strict oncological precautions.
